# A Re-Evaluation of the Effect of Trauma Center Verification Level on the Early Risk of Death in Hemodynamically Unstable Patients

**DOI:** 10.7759/cureus.14462

**Published:** 2021-04-13

**Authors:** David Plurad, Glenn Geesman, Nicholas Sheets, Bhani Chawla-Kondal, Ahmed Mahmoud

**Affiliations:** 1 Trauma and Acute Care Surgery, Riverside Community Hospital, Riverside, USA; 2 General Surgery, Riverside Community Hospital, Riverside, USA

**Keywords:** mortality, trauma center, verification, american college of surgeons, unstable trauma

## Abstract

Background

Studies show increased early and overall mortality at level II compared to level I trauma centers in hemodynamically unstable patients. We hypothesize there is no mortality difference between level I and level II centers applying more contemporary data.

Study design

Utilizing the 2017 Trauma Quality Program Participant Use File (TQP-PUF), we identified adult patients (age >14 years) who presented to an American College of Surgeons (ACS) verified level I or II center with hypotension (systolic blood pressure [SBP] < 90 mmHg). Logistic regression was performed to identify adjusted associations with mortality.

Results

A total of 7,264 patients met the inclusion criteria, of whom most were males (4,924 [67.8%]) with blunt trauma (5,924 [81.6%]) being predominated. Mean admission SBP was 73.2 (±13.0) mmHg. There were 1,097 (15.1%) deaths. Level I admissions (4,931 (67.9%]) were more likely male (3,389 [68.7%] vs. 1,535 [65.8]; p=0.012), non-white (3,119 [63.3%] vs. 1,664 [71.3%]; p<0.001), a victim of penetrating trauma (933 [18.9%] vs. 385 [16.5%]; p=0.015), and more severely injured (mean Injury Severity Score: 19.3 [±15] vs. 16.7 [±13.7]; p<0.001). Level II admissions (2,333 [32.1%]) were older (46.8 [±18.5] vs. 50.3 [±20.1] years; p<0.001) with more co-morbidities (mean Charlson Comorbidity Index: 1.43 [±2] vs. 1.77 [±2.2]; p<0.001). Adjusted mortality between level I and II admissions was similar (766 [15.5%] vs. 331 [14.2%]; p=0.918). Early hourly mortality also did not differ.

Conclusion

There is no overall or hourly mortality discrepancy between ACS-verified level I and II centers for patients presenting with hypotension. This potentially relates to the use of more contemporary data gathered after implementation of updated verification requirements.

## Introduction

Previous literature outlines significant outcome differences between level I and level II trauma centers. Superior outcomes are reported at level I centers for the severely injured [1,2], traumatic brain injury (TBI) patients [3,4], those with other specific injuries [5], and overall mortality [6,7]. Less prevalent are studies showing improved outcomes or equivalency at level II centers [8-11]. Of significance to our study, previous data show that trauma patients presenting with hemodynamic instability have significantly lower mortality in level I versus level II centers and that this discrepancy is sustained during the first hours of admission [2]. It was hypothesized that level II centers have access to inferior resources. However, during the time many of these investigations were being reported, there were differences in clinical requirements at level I versus level II trauma centers.

Since 1976, the American College of Surgeon Committee on Trauma (ACS-COT) has issued trauma center resource guidelines. “Resources for the Optimal Care of the Injured Patient” (resources manual) emphasizes the importance of a systems-based approach mandating escalating clinical resources at higher level trauma centers [12]. The 2014 update mandated equivalent clinical resources at level I and II centers so that, theoretically, outcomes would be similar. However, there are little recent data to support this. We hypothesize that more contemporary analysis would support improved outcomes at level II centers relative to their level I counterparts in patients who present with hemodynamic instability [2].

## Materials and methods

Utilizing the 2017 Trauma Quality Program Participant Use File (TQP-PUF), we identified adult patients (age >14 years) who presented to an ACS-COT verified level I or II trauma center with hemodynamic instability (systolic blood pressure [SBP] < 90 mmHg) [2]. We excluded patients with isolated TBI and interfacility transfers. Isolated TBI was identified by an Abbreviated Injury Scale (AIS) score for head of ≥3 with an AIS score for all other body regions of <3 [3]. We extracted all pertinent demographic and injury variables. This included, but was not limited to, gender, race, E code mechanism (mechanism), admission Glasgow Coma Scale (GCS) score, Injury Severity Score (ISS), and the presence of medical co-morbidities (Charlson Comorbidity Index [CCI]). Outcome variables include ICU and hospital lengths of stay (LOS) and in-hospital mortality.

Continuous variables were converted to dichotomous variables at clinically significant cut-points. This included, but not limited to, age (> 60 years), hypoxia (O_2_ saturation < 93%), severe TBI (admission GCS < 9), severe injury (ISS > 15), and CCI ≥ 3. Demographic and injury variables were compared between the groups admitted to a level I versus level II center. Similarly, variables were studied for their association with mortality. Univariate analysis was performed using Student’s t-test or ANOVA (analysis of variance) for continuous variables and X2 for dichotomous variables. All variables with a p-value of <0.05 on univariate analysis were then entered into logistic regression to determine adjusted mortality outcomes, with admission to a level II being added to the model. Results are reported as raw numbers, percentages, and odds ratios with 95% confidence intervals with p-values where appropriate. SPSS Version 21 (IBM Corp., Chicago, IL, USA) was used for statistical analysis. Comparisons were considered statistically significant with a p-value of <0.05.

## Results

**Figure 1:**
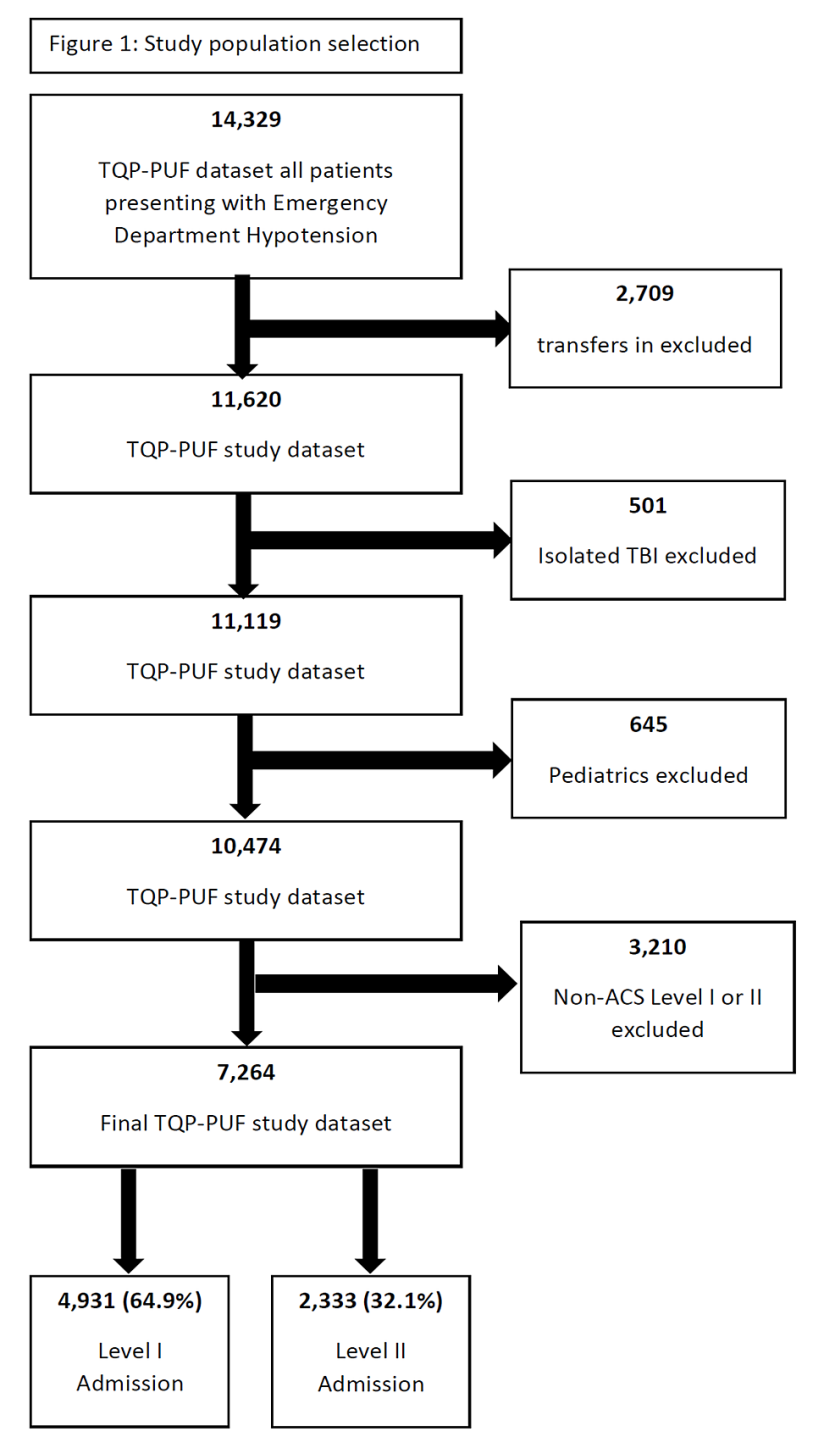
Study population selection TQP-PUF, Trauma Quality Program Participant Use File

There were 7,264 patients meeting the inclusion criteria (Figure 1). Most patients were male (4,924 [67.8%]) and white (4,783 [65.8%]). Mean age was 47.9 (± 19.5) years. Primary mechanisms were occupants in motor vehicle trauma (1,808 [24.9%]) followed by falls (1,346 [18.5%]). The study group was severely injured with a mean ISS of 18.5 (±14.6). Mean ICU and hospital LOS were 8 (±9.5) and 11.7 (±15.1) days, respectively. There were 1,097 (15.1%) in-hospital deaths (Table 1).

**Table 1:** Hypotensive (SBP < 90 mmHg) patients admitted to an ACS-COT level I or II trauma center Data are expressed as raw numbers, percentages, and means with standard deviations. ACS-COT, American College of Surgeon Committee on Trauma; CCI, Charlson Comorbidity Index; CHF, congestive heart failure; COPD, chronic obstructive pulmonary disease; CVA, cerebrovascular accident; ED, emergency department; HTN, hypertension; ICU, intensive care unit; ISS, Injury Severity Score; LOS, length of stay; MVT, motor vehicle traffic; OR, operating room; SBP, systolic blood pressure; TBI, traumatic brain injury

Variable	n or mean (% or SD)
Study patient	7,264
Level I admission	4,931 (67.9%)
Level II admission	2,333 (32.1%)
Demographics	
Gender (male)	4,924 (67.8%)
Mean age (years)	47.9 (±19.5)
Race	
Asian-Pacific Islander	147 (2.1%)
Black	748 (10.3%)
Other	1,586 (21.8%)
White	4738 (65.8%)
Mechanism (E code)	
Cut/pierce	737 (10.1%)
Fall	1,346 (18.5%)
Fall from height	556 (7.7%)
Firearm	1,096 (15.1%)
MVT-occupant	1,808 (25%)
MVT-motorcycle	553 (7.6%)
MVT-pedestrian	478 (6.7%)
Pedestrian/cyclist	276 (3.9%)
Struck by/against	236 (3.2%)
Other classifiable	143 (2.1%)
Trauma type	
Blunt	5,924 (81.6%)
Penetrating	1,318 (18.1%)
Thermal	22 (0.3%)
Injury severity/demographics	
Admission mean ISS	18.5 (±14.6)
ISS > 15	3,652 (50.3%)
Mean SBP (mmHg)	73.2 (±13)
Admission hypoxia (SpO_2_ < 93%)	
TBI all	2,032 (28%)
TBI mild	909 (12.5%)
TBI moderate	130 (1.8%)
TBI severe	993 (13.7%)
Co-morbidities	
Alcohol abuse disorder	757 (10.4%)
Anticoagulation	462 (6.4%)
CHF	276 (3.8%)
Cirrhosis	159 (2.2%)
COPD	467 (6.4%)
CVA	142 (2%)
Dementia	122 (1.7%)
Diabetes	846 (11.6%)
HTN	1,872 (25.8%)
Other	1,068 (14.7%)
Psychiatric disorder	939 (12.9%)
Renal dysfunction	131 (1.8%)
Substance abuse	783 (10.8%)
Chronic condition (any)	5,111 (70.4%)
Mean CCI	1.54 (±2.1)
Trauma center characteristics	
University teaching	4,066 (56%)
Community teaching	2,472 (34%)
Non-teaching	726 (10%)
<200 beds	419 (5.8%)
201–400 beds	2,013 (27.7%)
401–600 beds	1,978 (27.2%)
>600 beds	2,854 (39.3%)
Primary payor characteristics	
Medicare/Medicaid	3,189 (43.9%)
Private	2,566 (35.3%)
Other	344 (4.7%)
Uninsured	1,165 (16.1%)
Treatment after ED	
Ward	1,993 (27.4%)
OR	2,395 (33%)
ICU	2,722 (37.5%)
Death in ED	154 (2.1%)
Outcome	
ICU LOS (days)	8 (±9.8)
Hospital LOS (days)	11.7 (±13.9)
Deaths	1,097 (15.1%)

There were 4,921 (67.9%) patients admitted to a level I center, whereas 2,333 (32.1%) were treated at a level II center (Table 2). There was a slight male predominance (68.7% vs. 65.8%; p=0.012) at level I and the patients were less likely to be white (63.3% vs. 71.3%, <0.001). Level I admissions were also significantly younger (mean age: 46.8 [±19] vs. 50.3 [±20.1] years; p<0.001), with less comorbidities (mean CCI: 1.43 [±2] vs. 1.77 [±2.2]; p<0.001). Firearm injuries were more prevalent (16.9% vs. 11.3%; p<0.001). Level I admissions were more severely injured (mean ISS: 19.3 [±15] vs. 16.7 [±13.7]; p<0.001) and significantly more underwent surgery or angiography for hemorrhage control (not shown in table) (34.5% vs. 25.3%; p<0.001). The presence of a TBI was similar (27.8% vs. 28.3%; p=0.980). ICU LOS was not different but hospital LOS was longer at a level I center (mean 12.1 [±14] vs. 10.9 [±13.6] days; p<0.001). Mortality was higher at a Level I center; however, this was not statistically significant (15.5% vs. 14.2%, p=0.134).

**Table 2:** Comparison of admissions to level I versus level II trauma centers for hypotensive (SBP < 90 mmHg) patients Data are expressed as raw numbers, percentages, and means with standard deviations. CCI, Charlson Comorbidity Index; CHF, congestive heart failure; COPD, chronic obstructive pulmonary disease; CVA, cerebrovascular accident; ED, emergency department; HTN, hypertension; ICU, intensive care unit; ISS, Injury Severity Score; LOS, length of stay; MVT, motor vehicle traffic; OR, operating room; SBP, systolic blood pressure; TBI, traumatic brain injury

Variable	Level I: 4,931 (67.9%)	Level II: 2,333 (32.1%)	OR/mean difference (95% CI), p-value
Demographics			
Gender (male)	3,389 (68.7%)	1,535 (65.8%)	1.143 (1.029–­1.269), 0.012
Mean age (years)	46.8 (±19)	50.3 (±20.1)	–3.467 (–4.423 to 2.511), <0.001
Race			
Asian-Pacific Islander	111 (2.3%)	36 (1.5%)	<0.001
Black	476 (9.7%)	272 (11.7%)	
Other	1,225 (24.8%)	361 (15.5%)	
White	3,119 (63.3%)	1,664 (71.3%)	
Mechanism (E code)			
Cut/pierce	525 (10.6%)	212 (9.1%)	<0.001
Fall	781 (15.8%)	565 (24.2%)	
Fall from height	360 (7.3%)	196 (8.4%)	
Firearm	833 (16.9%)	263 (11.3%)	
MVT occupant	1,362 (25.6%)	546 (23.4%)	
MVT motorcycle	280 (7.7%)	173 (7.4%)	
MVT pedestrian	327 (6.6%)	151 (6.5%)	
Pedestrian/cyclist	187 (3.8%)	89 (3.8%)	
Struck by/against	151 (3.1%)	85 (3.6%)	
Other classifiable	101 (2%)	42 (1.8%)	
Trauma type			
Blunt	3,980 (80.7%)	1,944 (83.3%)	0.015
Penetrating	933 (18.9%)	385 (16.5%)	
Thermal	18 (0.4%)	4 (0.2%)	
Injury severity			
Mean ISS	19.3 (±15)	16.7 (±13.7)	2.611 (1.893–3.329), <0.001
ISS > 15	2,614 (53%)	1,038 (44.5%)	1.408 (1.275–1.554), <0.001
Admission mean SBP (mmHg)	73.4 (±18.5)	72.8 (±20)	0.637 (–0.300 to 1.574), 0.183
Admission hypoxia (SpO_2_ < 93%)	857 (42.8%)	420 (18%)	0.961 (0.845–1.093), 0.543
TBI all	1,372 (27.8%)	660 (28.3%)	0.977 (0.876–1.090), 0.980
TBI mild	587 (11.9%)	322 (13.8)	<0.001
TBI moderate	96 (1.9%)	34 (1.5%)	
TBI severe	689 (14%)	304 (13%)	
Co-morbidities			
Alcohol abuse disorder	515 (10.4%)	242 (10.4%)	1.008 (0.858–1.184), 0.929
Anticoagulation	294 (6%)	168 (7.2%)	0.817 (0.671–0.994), 0.043
CHF	180 (3.7%)	96 (4.1%)	0.883 (0.686–1.137), 0.334
Cirrhosis	116 (2.4%)	43 (1.8%)	1.283 (0.901–1.827), 0.166
COPD	306 (6.2%)	161 (6.9%)	0.893 (0.733–1.087), 0.259
CVA	85 (1.7%)	57 (2.4%)	0.700 (0.499–0.983), 0.039
Dementia	74 (1.5%)	48 (2.1%)	0.725 (0.503–1.046), 0.085
Diabetes	523 (10.6%)	323 (13.8%)	0.738 (0.637–0.856), <0.001
HTN	1,171 (23.7%)	701 (30%)	0.725 (0.649–0.809), <0.001
Other	700 (14.2%)	368 (15.8%)	0.770 (1.013–1.013), 0.076
Psychiatric disorder	652 (13.2%)	287 (12.3%)	1.086 (0.936–1.260), 0.275
Renal dysfunction	89 (1.8%)	42 (1.8%)	1.003 (0.692–1.452), 0.989
Substance abuse	561 (11.4%)	222 (9.5%)	1.221 (1.036–1.438), 0.017
Chronic condition (any)	3,418 (69.3%)	1,693 (72.6%)	0.854 (0.766–0.953), 0.005
Mean CCI	1.43 (±2)	1.77 (±2.2)	0.695 (0.623–0.773), <0.001
Trauma center characteristics			
University teaching	3,802 (77.1%)	279 (12%)	<0.001
Community teaching	1,139 (22.9%)	1,343 (57.3%)	
Non-teaching	0 (0%)	711 (30.5%)	
<200 beds	202 (4.1%)	217 (9.3%)	<0.001
201–400 beds	631 (12.8%)	1,382 (59.2%)	
401–600 beds	1,625 (33%)	353 (15.1%)	
>600 beds	2,473 (50.2%)	381 (16.3%)	
Primary payor characteristics			
Medicare/Medicaid	2,148 (43.6%)	1,041 (44.6%)	<0.001
Private insurance	1,651 (33.5%)	915 (39.2%)	
Uninsured	901 (18.3%)	264 (11.3%)	
Other	231 (4.7%)	113 (4.8%)	
Treatment after ED			
Ward	1,274 (25.8%)	719 (30.8%)	<0.001
OR	1,700 (34.5%)	695 (29.8%)	
ICU	1,842 (37.4%)	880 (37.7%)	
Death in ED	115 (2.3%)	39 (1.7%)	1.405 (0.974–2.026), 0.081
Outcomes			
ICU LOS (days)	7.9 (±9.2)	8.2 (±10.2)	–0.262 (–0.843 to 0.318), 0.376
Hospital LOS (days)	12.1 (±14)	10.9 (±13.6)	1.269 (0.587–1.951), <0.001
Mortality	766 (15.5%)	331 (14.2%)	1.112 (0.968–1.279), 0.134

Deaths are compared to survivors in Table 3. Males (73.2% vs. 66.8%; p<0.001) and victims of firearm injuries (20.4% vs. 14.1%, <0.001) were more likely to die. The presence of a TBI (57.1% vs. 23%, <0.001) and higher mean ISS (32.6 [±17.1] vs. 16 [±12.6], p<0.001) were also associated with increased mortality. Increasing instability, reflected in lower mean admission SBP, also predicted death (mean SBP: 60.3 [±29.6] vs. 75.5 [±15.4] mmHg; p<0.001). Co-morbidities (CCI: 1.64 (±2.28) vs. 1.52 [2.02]; p=0.081)) were not statistically associated with death on univariate analysis, though age > 60 years (34.1% vs. 29.7%; p=0.003) predicted mortality.

**Table 3:** Comparison of deaths and survivors for hypotensive (SBP < 90 mmHg) trauma patients Data are expressed as raw numbers, percentages, and means with standard deviations. CCI, Charlson Comorbidity Index; CHF, congestive heart failure; COPD, chronic obstructive pulmonary disease; CVA, cerebrovascular accident; HTN, hypertension; ISS, Injury Severity Score; MVT, motor vehicle traffic; SBP, systolic blood pressure; TBI, traumatic brain injury

Variable	Death: 1,097 (15.1%)	Survivor: 6,167 (84.9%)	OR/mean difference (95% CI), p-value
Demographics			
Gender (male)	803 (73.2%)	4,121 (66.8%)	1.356 (1.174–1.566), <0.001
Mean age (years)	48.1 (±21.5)	47 (±19.1)	0.216 (–1.035 to 1.466), 0.735
Age > 60 years	374 (34.1%)	1,830 (29.7%)	1.226 (1.070–1.405), 0.003
Race			
Asian-Pacific Islander	24 (2.2%)	123 (2%)	0.924
Black	108 (9.8%)	640 (10.4%)	
Other	238 (21.9%)	1,348 (21.9%)	
White	727 (66.3%)	4,056 (65.8%)	
Mechanism (E code)			
Cut/pierce	44 (4%)	693 (11.2%)	<0.001
Fall	101 (9.2%)	1,245 (20.2%)	
Fall from height	84 (7.7%)	472 (7.7%)	
Firearm	224 (20.4%)	872 (14.1%)	
MVT occupant	316 (28.8%)	1,492 (24.2%)	
MVT motorcycle	110 (10%)	443 (7.2%)	
MVT pedestrian	105 (9.6%)	373 (6%)	
Pedestrian/cyclist	50 (4.6%)	226 (3.7%)	
Struck by/against	22 (2%)	214 (3.4%)	
Other classifiable	21 (2.8%)	111 (1.8%)	
Trauma type			
Blunt	903 (82.3%)	5,021 (81.4%)	0.480
Penetrating	188 (17.1%)	1,130 (18.3%)	
Thermal	6 (0.5%)	16 (0.3%)	
Injury severity			
Mean ISS	32.6 (±17.1)	16 (±12.6)	16.606 (15.748–17.465), <0.001
ISS > 15	960 (87.5%)	2,692 (43.7%)	9.045 (7.511–10.894), <0.001
Admission mean SBP (mmHg)	60.3 (±39.6)	75.5 (±15.4)	–15.253 (–16.424 to 14.083), <0.001
Admission Hypoxia (SpO_2_ < 93%)	375 (34.2%)	904 (14.7%)	3.024 (2.620–3.490), <0.001
TBI all	626 (57.1%)	1,416 (23%)	4.297 (3.761–4.909), <0.001
TBI mild	70 (6.4%)	839 (13.6%)	<0.001
TBI moderate	11 (1%)	119 (1.9%)	
TBI severe	532 (48.5%)	458 (7.4%)	
Co-morbidities			
Alcohol abuse disorder	64 (5.8%)	693 (11.2%)	0.489 (0.376–0.638), <0.001
Anticoagulation	57 (5.2%)	405 (6.6%)	0.780 (0.586–1.037), 0.086
CHF	39 (3.6%)	237 (3.8%)	0.922 (0.653–1.302), 0.646
Cirrhosis	43 (3.9%)	116 (1.9%)	2.128 (1.491–3.038), <0.001
COPD	47 (4.3%)	420 (6.8%)	0.612 (0.450–0.834), 0.002
CVA	11 (1%)	131 (2.1%)	0.467 (0.251–0.866), 0.013
Dementia	18 (1.6%)	104 (1.7%)	0.973 (0.587–1.611), 0.914
Diabetes	89 (8.1%)	757 (12.3%)	0.631 (0.502–0.794), <0.001
HTN	193 (17.6%)	1,679 (27.2%)	0.571 (0.484–0.673), 0.001
Other	153 (13.9%)	915 (14.8%)	0.930 (0.773–1.119), 0.443
Psychiatric disorder	85 (7.7%)	854 (13.8%)	0.523 (0.414–0.660), <0.001
Renal dysfunction	26 (2.4%)	105 (1.7%)	1.402 (0.908–2.164), 0.126
Substance abuse	59 (5.4%)	724 (11.7%)	0.427 (0.325–0.532), <0.001
Chronic condition (any)	670 (61.1%)	4,441 (72%)	0.610 (0.534–0.697), <0.001
Mean CCI	1.64 (±2.28)	1.52 (±2.02)	0.118 (–0.014 to 0.250), 0.081
Trauma center characteristics			
Level I trauma center	766 (69.8%)	4,165 (67.5%)	1.112 (0.968–1.279), 0.141
University teaching	657 (59.9%)	3,424 (55.5%)	<0.001
Community teaching	343 (31.3%)	2,129 (34.6%)	
Non-teaching	97 (8.8%)	614 (10%)	
<200 beds	66 (6%)	353 (5.7%)	<0.001
201–400 beds	282 (25.7%)	1,731 (28.1%)	
401–600 beds	291 (26.5%)	1,687 (27.4%)	
>600 beds	458 (41.8%)	2,396 (38.9%)	
Primary payor characteristics			
Medicare/Medicaid	396 (36.1%)	2,793 (45.3%)	<0.001
Private insurance	362 (33%)	2,204 (35.7%)	
Uninsured	280 (25.5%)	885 (14.4%)	
Other	59 (5.4%)	285 (4.6%)	

**Figure 2:**
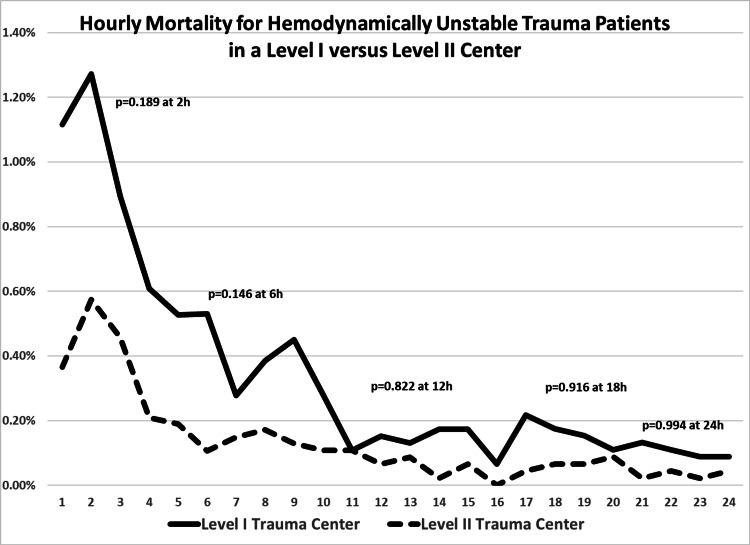
Hourly mortality for hemodynamically unstable trauma patients in a level I versus level II center

On logistic regression (Table 4), admission GCS < 9, ISS > 15, age > 60 years, hypoxia, payor group, mechanism, and the presence of a TBI were independently associated with in-hospital death, but admission to a level I versus a level II center was not (1.009 [0.851-1.196]; p=0.918). The hourly risk of death, similarly, was not statistically significant with admission to a level I versus II center (Figure 2).

**Table 4:** Adjusted mortality outcomes for hypotensive trauma patients Other variables entered into forward stepwise regression: gender, race, CCI, hospital teaching type, hospital size GCS Glasgow Coma Scale; ISS, Injury Severity Score; TBI, traumatic brain injury

Variable	Exp(B) (95% CI for Exp(B)), p-value
Level I versus level II	1.009 (0.851–1.196), 0.918
GCS < 9	10.806 (9.019–12.947), <0.001
ISS > 15	4.508 (3.638–5.590), <0.001
Age > 60 years	3.821 (3.142–4.646), <0.001
Hypoxia (SpO_2_ < 93%)	1.901 (1.596–2.264), <0.001
Primary payor	0.799 (0.729–0.876), <0.001
Mechanism (E Code)	1.44 (1.167–1.778), 0.001
TBI any	1.323 (1.110–1.577), 0.002

## Discussion

Literature is replete with studies addressing the outcome differences between level I and II trauma centers. Significant mortality increases at level II centers for specific subsets have been demonstrated. These include the severely injured [1,2], those with specific wounding [5], those with TBI [8,11], those transferred to a level II center after TBI [13], and overall populations [6,7]. Of specific interest to our study, those admitted with hemodynamic instability have been shown to have better mortality outcomes at a level I center that is sustained through early admission [2]. Of concern is that these reports may utilize obsolete data gathered prior to the 2014 “resources manual” update [12]. The previously mentioned study [2] on hypotensive trauma patients is an example of one of the more egregious confounding as it utilized data nearly over a decade old to support conclusions that were no longer valid at the time of publication.

The 2014 revision of the ACS-COT resources manual (Orange Book) specifically addresses clinical resourcing discrepancies between level I and II ACS-COT verified centers. “Level I and II criteria were revised to ensure that level I and II trauma centers are available to provide high quality definitive care” [12]. The assumption is that this would lead to reduction or elimination outcome discrepancies, though there are little data to support this. Our current investigation demonstrates this relative improvement and contradicts earlier reports. Furthermore, previous studies that demonstrate outcome equivalence or even improved outcomes at level II centers used locoregional data [8,14].

Our investigation shows no mortality difference between admission to a level I versus level II center in patients who present with hemodynamic instability. We hypothesize that this is associated with utilization of more contemporary datasets that were gathered after implementation of the 2014 ACS-COT “resources manual”. Updated requirements include, but are not limited to, uninterrupted emergency medicine staffing, more stringent operating room resourcing with performance tracking, defined minimums for highest level activations, minimum registrar training requirements, dedicated injury prevention positions, equivalent surgical and non-surgical subspecialty services, and changes in guidance for consultant bedside presence. One of the more pertinent improvements for both level I and II centers relate to performance improvement mandating exacting identification and trending of important outcome and process metrics. In addition, participation in a risk-adjusted outcome benchmarking program (Trauma Quality Improvement Program [TQIP]) was a significant new mandate [15]. Elements for a level I center, not required of a level II center, are admission volume minimums, the presence of higher level surgical resident trainees, a surgically directed intensive care unit, and minimum research productivity.

Despite a reasonably valid clinical association between improved resources and better outcomes, our study is hindered by its retrospective design. Trauma care at level II centers could have simply improved over time with the introduction of new techniques and protocols, though this is doubtful relative to outcome improvements related to mandated improved resourcing. While the 2017 TQP-PUF is a powerful tool to assess the impact of sweeping administrative mandates, it lacks the granularity to define elements that may have had an impact on our findings or that could have contributed to confounding. There were significant differences between level I and level II admissions. Most notably, level I admissions were more severely injured and differed demographically and by mechanism. Despite significant differences, adjusted mortality that included these variables was similar between level I and II centers and sustained hourly through the first 24 hours of admission. Of note, co-morbidities were not associated with death. This could possibly be due to survivors having more opportunity for their care teams to identify and document co-morbidities.

Applicability of our study extends only to ACS-COT verified centers regardless of the designating authority. While the scope of this organization is broad and many local designating authorities model their verification efforts to mirror the ACS-COT process and requirements, not all designated trauma centers are verified by this committee. It may be reasonable to assume that level II trauma centers verified by their local designating authority or other non-ACS-COT construct also experience improved outcomes relative to level I; however, our study excluded these trauma centers.

Despite these shortcomings, our study is impactful since it demonstrates, in contradiction to older published data, that level II trauma centers achieve similar outcomes in patients who present with hemodynamic instability compared to level I centers. In this subgroup, immediate presence of trauma surgeons, competent consultants, and timely availability of interventions are critical [15]. This change likely relates, in part, to the updated requirements that these elements be in place at a level II center just as they are at the level I center. Additionally, our study demonstrates the potentially significant and widespread beneficial impact of ongoing process improvement, resource standardization, and involvement of a national verification program. It is important for the public and policy-makers since it supports that the significant investment in a level II can be expected to generate outcomes similar to those at a level I center.

## Conclusions

As opposed to previous studies, level II trauma centers perform similar to level I centers for trauma patients who present with hemodynamic instability. This may relate to compliance with the ACS-COT resources manual, Resources for the Optimal Care of the Injured Patient, 2014 version. Further study would include examination of trauma centers that are not verified by the ACS-COT that could reveal more specific variables associated with improved outcome in these patients.
